# Materia Medica and Therapeutics

**Published:** 1896-01

**Authors:** 


### Book Notices.

MATERIA MEDICA AND THERAPEUTICS. A Practical Treatise with Especial Reference to the Clinical Application of Drugs. By John V. Shoemaker, A. M., M. D., LL. D., Professor of Materia Medica, Pharmacology, Therapeutics, and Clinical Medicine, and Clinical Professor of Diseases of the Skin in the Medico-Chirurgical College of Philadelphia; Physician to the Medico-Chirurgical Hospital, Philadelphia, etc., etc. Third edition, thoroughly revised. Reset with new type and printed from new electrotype plates. Royal octavo, pages, 1108. Extra cloth, $5 net; sheep, $5.75. Philadelphia: The F. A. Davis Co., publishers, 1914 and 1916 Cherry street.

Only two years ago the second edition of Dr. Shoemaker's Materia Medica and Therapeutics was issued, but owing to the many new preparations introduced to the profession, and the many new applications of the older remedies discovered during these two years, and encouraged by the very favorable reception given the former edition by the profession, a third edition became necessary. In this edition many new compounds have been introduced for the first time, including tolysal, tolypyrin, salocoll, salocetol, chlorphenol, bromphenol, tropococaine, formaldehyde, and formalin, dulcin, tannigen, etc.

The present edition is issued in one volume of over 1100 pages, and the list of subjects embrace not only pharmaceutical therapeutic agents, but twenty-eight pages are given to pharmacy, twenty-three to prescription writing and formulæ, three to poisons and antidotes, thirteen to general therapeutics and classification of remedies, seventy to electricity in medicine, eighteen to massage, besides a number of pages to hydrotherapy, climatology, hypnotism, various mechanical appliances, local therapeutics, etc. This volume has two complete indices, a general and a clinical, also a table of doses and formulæ for hypodermic use, and for preparing a suitable diet for the sick. It is complete and at the same time short enough to be eminently practical, and is abreast of the present status of materia-medica and therapeutics. H.

